# (2-Benzoyl­phen­yl)(naphthalen-1-yl)methanone

**DOI:** 10.1107/S1600536812039098

**Published:** 2012-09-26

**Authors:** G. Ganesh, R. Sivasakthikumaran, E. Govindan, A. K. Mohana Krishnan, A. SubbiahPandi

**Affiliations:** a Department of Physics, S.M.K. Fomra Institute of Technology, Thaiyur, Chennai 603 103, India; b Department of Organic Chemistry, University of Madras, Guindy Campus, Chennai 600 025, India; c Department of Physics, Presidency College (Autonomous), Chennai 600 005, India

## Abstract

In the title compound, C_24_H_16_O_2_, the naphthalene ring system makes dihedral angles of 78.5 (6) and 65.5 (7)° with the terminal and central benzene rings, respectively. The dihedral angle between the benzene rings is 74.5 (8)°. In the crystal, neighbouring molecules are interlinked through two C—H⋯π interactions, which construct a two-dimensional supramolecular framework extending infinitely along (010).

## Related literature

For the biological activity of naphthalene derivatives, see: Wiltz *et al.* (1998 ▶); Wright *et al.* (2000 ▶); Varma *et al.* (1994 ▶). For a related structure, see: Xia (2010 ▶).
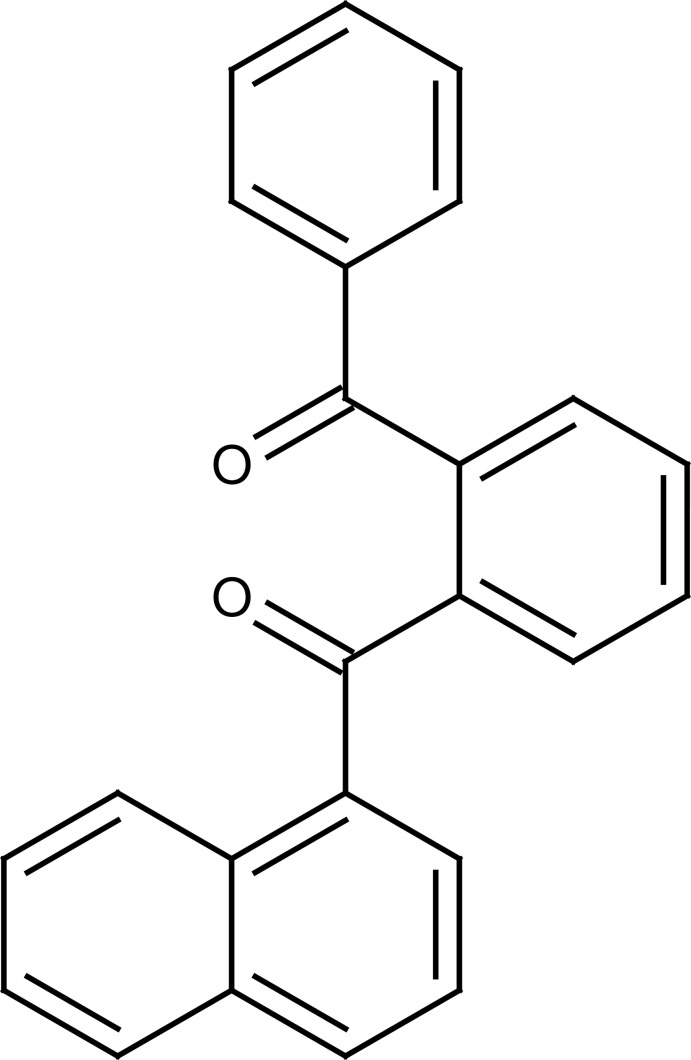


## Experimental

### 

#### Crystal data

C_24_H_16_O_2_
*M* *_r_* = 336.37
Monoclinic, 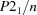

*a* = 10.4105 (3) Å
*b* = 9.6218 (3) Å
*c* = 17.8497 (5) Å
β = 106.113 (2)°
*V* = 1717.73 (9) Å^3^
*Z* = 4
Mo *K*α radiation
μ = 0.08 mm^−1^
*T* = 293 K
0.25 × 0.22 × 0.19 mm

#### Data collection

Bruker APEXII CCD area-detector diffractometer
Absorption correction: multi-scan (*SADABS*; Sheldrick, 1996 ▶) *T*_min_ = 0.980, *T*_max_ = 0.985
18980 measured reflections
4081 independent reflections
2906 reflections with *I* > 2σ(*I*)
*R*_int_ = 0.024

#### Refinement

*R*[*F* ^2^ > 2σ(*F* ^2^)] = 0.043
*wR*(*F* ^2^) = 0.135
*S* = 1.01
4081 reflections
235 parameters
H-atom parameters constrained
Δρ_max_ = 0.22 e Å^−3^
Δρ_min_ = −0.17 e Å^−3^

### 

Data collection: *APEX2* (Bruker, 2008 ▶); cell refinement: *SAINT* (Bruker, 2008 ▶); data reduction: *SAINT*; program(s) used to solve structure: *SHELXS97* (Sheldrick, 2008 ▶); program(s) used to refine structure: *SHELXL97* (Sheldrick, 2008 ▶); molecular graphics: *ORTEP-3* (Farrugia, 1997 ▶); software used to prepare material for publication: *SHELXL97* and *PLATON* (Spek, 2009 ▶).

## Supplementary Material

Crystal structure: contains datablock(s) global, I. DOI: 10.1107/S1600536812039098/bt6830sup1.cif

Structure factors: contains datablock(s) I. DOI: 10.1107/S1600536812039098/bt6830Isup2.hkl

Supplementary material file. DOI: 10.1107/S1600536812039098/bt6830Isup3.cml

Additional supplementary materials:  crystallographic information; 3D view; checkCIF report

## Figures and Tables

**Table 1:** Hydrogen-bond geometry (Å, °) *Cg*1 and *Cg*2 are the centroids of the C1–C6 and C8–C13 rings, respectively.

*D*—H⋯*A*	*D*—H	H⋯*A*	*D*⋯*A*	*D*—H⋯*A*
C11—H11⋯*Cg*1^i^	0.93	2.71	3.618 (19)	163
C20—H20⋯*Cg*2^ii^	0.93	2.85	3.67 (3)	141

Symmetry codes: (i) 
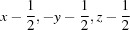
; (ii) 
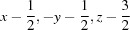
.

**Fig. 1.**
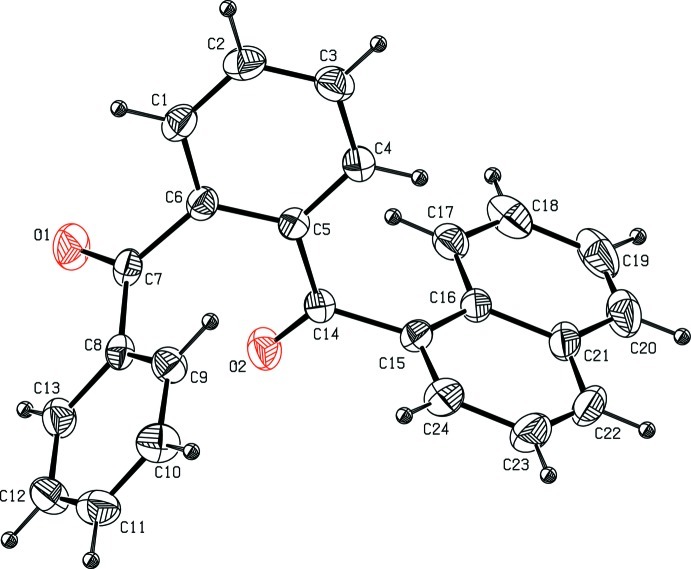
The structure of showing the atom-numbering scheme. The displacement ellipsoids are drawn at the 30% probability level.

## supplementary crystallographic information

### Comment

Naphthalene derivatives have manifested applications in many fields, for example, as a colorant, explosive, disinfectant, insecticide and plant hormone auxin. Naphthalene is believed to play a role in the chemical defence against biological enemies (Wiltz *et al.*, 1998; Wright *et al.*, 2000). It may be produced by metabolic processes in termites or by associated microorganisms which inhabit, *e.g.*, the termite guts (Varma *et al.*, 1994).

Bond lengths and bond angles of the title compound are comparable with the related structure (Xia, 2010). The napthyl ring C15—C24 system makes dihedral angles of 78.5 (6)° and 65.5 (7)°, with the phenyl rings C1—C6 and the C8—C13 respectively.

The C–H···π interaction C11–H11—Cg1i (Cg1 : C1–C6) [symmetry code : (i) x-1/2, -y-1/2, z-1/2] links the adjacent title moleculesto generate an extended one dimensional supramolecular network along [104] direction. Parallely stacked [104] network are further linked through C20–H20···Cg2ii (Cg2 : C8–C13) [symmetry code : (i) x-1/2, -y-1/2, z-3/2] interaction to form a two dimensional supramolecular sheet extending parallel to the (010) plane.

### Experimental

To a stirred suspension of benzo[*c*]furan (2.38 g, 7.437 mmol) in dry THF (20 ml), lead tetraacetate (3.2 g, 7.437 mmol) was added and refluxed at 50°C for half an hour. The reaction mixture was then poured into water (200 ml) and extracted with ethyl acetate (2 *x* 20 ml), washed with brine solution and dried (Na_2_SO_4_). The removal of solvent *in vacuo* followed by crystallization from methanol afforded the title compound as a colorless solid. Single crystals suitable for X-ray diffraction were obtained by slow evaporation of a solution of the title compound in methanol at room temperature.

### Refinement

All H atoms were fixed geometrically and allowed to ride on their parent C atoms, with C—H distances fixed at 0.93Å with *U*_iso_(H) = 1.2*U*_eq_(C).

### Crystal data

**Table:**

C_24_H_16_O_2_	*F*(000) = 704
*M**_r_* = 336.37	*D*_x_ = 1.301 Mg m^−^^3^
Monoclinic, *P*2_1_/*n*	Mo *K*α radiation, λ = 0.71073 Å
Hall symbol: -P 2yn	Cell parameters from 4106 reflections
*a* = 10.4105 (3) Å	θ = 2.1–27.9°
*b* = 9.6218 (3) Å	µ = 0.08 mm^−^^1^
*c* = 17.8497 (5) Å	*T* = 293 K
β = 106.113 (2)°	Block, white crystalline
*V* = 1717.73 (9) Å^3^	0.25 × 0.22 × 0.19 mm
*Z* = 4	

### Data collection

**Table:**

Bruker APEXII CCD area-detector diffractometer	4081 independent reflections
Radiation source: fine-focus sealed tube	2906 reflections with *I* > 2σ(*I*)
Graphite monochromator	*R*_int_ = 0.024
ω and φ scans	θ_max_ = 27.9°, θ_min_ = 2.1°
Absorption correction: multi-scan (*SADABS*; Sheldrick, 1996)	*h* = −13→13
*T*_min_ = 0.980, *T*_max_ = 0.985	*k* = −9→12
18980 measured reflections	*l* = −23→22

### Refinement

**Table:**

Refinement on *F*^2^	Primary atom site location: structure-invariant direct methods
Least-squares matrix: full	Secondary atom site location: difference Fourier map
*R*[*F*^2^ > 2σ(*F*^2^)] = 0.043	Hydrogen site location: inferred from neighbouring sites
*wR*(*F*^2^) = 0.135	H-atom parameters constrained
*S* = 1.01	*w* = 1/[σ^2^(*F*_o_^2^) + (0.0682*P*)^2^ + 0.3208*P*] where *P* = (*F*_o_^2^ + 2*F*_c_^2^)/3
4081 reflections	(Δ/σ)_max_ < 0.001
235 parameters	Δρ_max_ = 0.22 e Å^−^^3^
0 restraints	Δρ_min_ = −0.17 e Å^−^^3^

### Special details

**Table:**

Geometry. All e.s.d.'s (except the e.s.d. in the dihedral angle between two l.s. planes) are estimated using the full covariance matrix. The cell e.s.d.'s are taken into account individually in the estimation of e.s.d.'s in distances, angles and torsion angles; correlations between e.s.d.'s in cell parameters are only used when they are defined by crystal symmetry. An approximate (isotropic) treatment of cell e.s.d.'s is used for estimating e.s.d.'s involving l.s. planes.
Refinement. Refinement of *F*^2^ against ALL reflections. The weighted *R*-factor *wR* and goodness of fit *S* are based on *F*^2^, conventional *R*-factors *R* are based on *F*, with *F* set to zero for negative *F*^2^. The threshold expression of *F*^2^ > σ(*F*^2^) is used only for calculating *R*-factors(gt) *etc*. and is not relevant to the choice of reflections for refinement. *R*-factors based on *F*^2^ are statistically about twice as large as those based on *F*, and *R*- factors based on ALL data will be even larger.

### Fractional atomic coordinates and isotropic or equivalent isotropic displacement parameters (Å^2^)

**Table:**

	*x*	*y*	*z*	*U*_iso_*/*U*_eq_	
C1	−0.12964 (14)	0.43758 (18)	0.19247 (9)	0.0514 (4)	
H1	−0.1978	0.4398	0.2168	0.062*	
C2	−0.11530 (16)	0.54645 (18)	0.14567 (10)	0.0577 (4)	
H2	−0.1746	0.6209	0.1379	0.069*	
C3	−0.01362 (17)	0.54578 (18)	0.11029 (10)	0.0572 (4)	
H3	−0.0030	0.6205	0.0795	0.069*	
C4	0.07240 (15)	0.43421 (16)	0.12060 (9)	0.0501 (4)	
H4	0.1413	0.4341	0.0967	0.060*	
C5	0.05740 (13)	0.32213 (15)	0.16614 (8)	0.0410 (3)	
C6	−0.04434 (13)	0.32506 (15)	0.20384 (8)	0.0417 (3)	
C7	−0.06077 (13)	0.21477 (16)	0.25979 (8)	0.0451 (3)	
C8	0.04444 (13)	0.20243 (14)	0.33523 (8)	0.0404 (3)	
C9	0.14254 (15)	0.30231 (16)	0.35969 (8)	0.0482 (3)	
H9	0.1457	0.3780	0.3278	0.058*	
C10	0.23568 (17)	0.28980 (18)	0.43124 (10)	0.0598 (4)	
H10	0.3014	0.3574	0.4475	0.072*	
C11	0.23221 (18)	0.1786 (2)	0.47854 (10)	0.0635 (5)	
H11	0.2953	0.1708	0.5268	0.076*	
C12	0.13550 (18)	0.0788 (2)	0.45457 (10)	0.0641 (5)	
H12	0.1337	0.0028	0.4864	0.077*	
C13	0.04130 (16)	0.09075 (17)	0.38380 (9)	0.0534 (4)	
H13	−0.0250	0.0236	0.3683	0.064*	
C14	0.14196 (13)	0.19692 (15)	0.17171 (8)	0.0423 (3)	
C15	0.26477 (13)	0.20331 (15)	0.14345 (9)	0.0453 (3)	
C16	0.25771 (14)	0.16839 (14)	0.06570 (9)	0.0452 (3)	
C17	0.13610 (17)	0.13546 (16)	0.00976 (9)	0.0524 (4)	
H17	0.0561	0.1393	0.0233	0.063*	
C18	0.1356 (2)	0.09810 (19)	−0.06394 (10)	0.0701 (5)	
H18	0.0556	0.0751	−0.1004	0.084*	
C19	0.2565 (3)	0.0944 (2)	−0.08489 (13)	0.0841 (7)	
H19	0.2556	0.0689	−0.1353	0.101*	
C20	0.3734 (3)	0.1272 (2)	−0.03286 (14)	0.0783 (6)	
H20	0.4520	0.1247	−0.0480	0.094*	
C21	0.37831 (17)	0.16475 (16)	0.04334 (11)	0.0579 (4)	
C22	0.49996 (17)	0.1967 (2)	0.10048 (15)	0.0723 (6)	
H22	0.5797	0.1944	0.0867	0.087*	
C23	0.50251 (17)	0.2301 (2)	0.17366 (14)	0.0743 (6)	
H23	0.5835	0.2503	0.2099	0.089*	
C24	0.38446 (16)	0.2346 (2)	0.19557 (11)	0.0646 (5)	
H24	0.3870	0.2592	0.2463	0.078*	
O1	−0.16245 (11)	0.14676 (16)	0.24595 (7)	0.0731 (4)	
O2	0.11301 (12)	0.08900 (12)	0.19794 (7)	0.0604 (3)	

### Atomic displacement parameters (Å^2^)

**Table:**

	*U*^11^	*U*^22^	*U*^33^	*U*^12^	*U*^13^	*U*^23^
C1	0.0423 (7)	0.0617 (10)	0.0521 (8)	0.0068 (7)	0.0164 (6)	−0.0069 (7)
C2	0.0572 (9)	0.0518 (10)	0.0615 (9)	0.0156 (7)	0.0124 (7)	−0.0009 (7)
C3	0.0655 (10)	0.0477 (9)	0.0583 (9)	0.0072 (7)	0.0172 (8)	0.0105 (7)
C4	0.0507 (8)	0.0525 (9)	0.0517 (8)	0.0047 (7)	0.0218 (7)	0.0079 (7)
C5	0.0373 (6)	0.0465 (8)	0.0399 (7)	0.0021 (6)	0.0120 (5)	0.0020 (6)
C6	0.0360 (6)	0.0502 (8)	0.0388 (7)	−0.0014 (6)	0.0103 (5)	−0.0032 (6)
C7	0.0365 (6)	0.0554 (9)	0.0476 (8)	−0.0056 (6)	0.0185 (6)	−0.0028 (6)
C8	0.0412 (7)	0.0426 (8)	0.0425 (7)	−0.0005 (6)	0.0200 (6)	−0.0029 (6)
C9	0.0515 (8)	0.0450 (8)	0.0476 (8)	−0.0047 (6)	0.0131 (6)	0.0007 (6)
C10	0.0584 (9)	0.0552 (10)	0.0583 (9)	−0.0065 (8)	0.0038 (7)	−0.0067 (8)
C11	0.0658 (10)	0.0691 (12)	0.0497 (9)	0.0113 (9)	0.0062 (8)	0.0034 (8)
C12	0.0744 (11)	0.0610 (11)	0.0599 (10)	0.0090 (9)	0.0238 (8)	0.0186 (8)
C13	0.0558 (8)	0.0488 (9)	0.0608 (9)	−0.0050 (7)	0.0246 (7)	0.0036 (7)
C14	0.0434 (7)	0.0462 (8)	0.0394 (7)	0.0016 (6)	0.0153 (6)	0.0020 (6)
C15	0.0395 (7)	0.0442 (8)	0.0551 (8)	0.0067 (6)	0.0178 (6)	0.0075 (6)
C16	0.0506 (8)	0.0354 (7)	0.0574 (8)	0.0096 (6)	0.0281 (7)	0.0095 (6)
C17	0.0641 (9)	0.0451 (8)	0.0517 (8)	0.0049 (7)	0.0221 (7)	0.0061 (7)
C18	0.1059 (15)	0.0519 (10)	0.0538 (9)	0.0014 (10)	0.0243 (10)	0.0047 (8)
C19	0.149 (2)	0.0582 (12)	0.0668 (12)	0.0200 (13)	0.0655 (14)	0.0093 (9)
C20	0.1069 (16)	0.0585 (12)	0.0952 (15)	0.0211 (11)	0.0707 (14)	0.0163 (10)
C21	0.0665 (10)	0.0403 (8)	0.0830 (11)	0.0154 (7)	0.0474 (9)	0.0164 (8)
C22	0.0474 (9)	0.0612 (11)	0.1210 (17)	0.0115 (8)	0.0445 (10)	0.0251 (11)
C23	0.0413 (8)	0.0803 (14)	0.0990 (15)	0.0006 (9)	0.0156 (9)	0.0176 (11)
C24	0.0485 (8)	0.0747 (12)	0.0677 (11)	0.0003 (8)	0.0114 (8)	0.0080 (9)
O1	0.0483 (6)	0.0960 (10)	0.0732 (8)	−0.0269 (6)	0.0139 (5)	0.0079 (7)
O2	0.0730 (7)	0.0453 (6)	0.0755 (7)	0.0027 (5)	0.0415 (6)	0.0069 (5)

### Geometric parameters (Å, º)

**Table:**

C1—C2	1.374 (2)	C12—H12	0.9300
C1—C6	1.379 (2)	C13—H13	0.9300
C1—H1	0.9300	C14—O2	1.2110 (17)
C2—C3	1.375 (2)	C14—C15	1.5001 (18)
C2—H2	0.9300	C15—C24	1.366 (2)
C3—C4	1.377 (2)	C15—C16	1.410 (2)
C3—H3	0.9300	C16—C17	1.414 (2)
C4—C5	1.385 (2)	C16—C21	1.420 (2)
C4—H4	0.9300	C17—C18	1.362 (2)
C5—C6	1.4039 (18)	C17—H17	0.9300
C5—C14	1.479 (2)	C18—C19	1.410 (3)
C6—C7	1.499 (2)	C18—H18	0.9300
C7—O1	1.2101 (17)	C19—C20	1.347 (3)
C7—C8	1.486 (2)	C19—H19	0.9300
C8—C9	1.382 (2)	C20—C21	1.395 (3)
C8—C13	1.387 (2)	C20—H20	0.9300
C9—C10	1.378 (2)	C21—C22	1.422 (3)
C9—H9	0.9300	C22—C23	1.338 (3)
C10—C11	1.370 (3)	C22—H22	0.9300
C10—H10	0.9300	C23—C24	1.390 (2)
C11—C12	1.371 (3)	C23—H23	0.9300
C11—H11	0.9300	C24—H24	0.9300
C12—C13	1.372 (2)		
			
C2—C1—C6	120.92 (14)	C12—C13—C8	120.36 (15)
C2—C1—H1	119.5	C12—C13—H13	119.8
C6—C1—H1	119.5	C8—C13—H13	119.8
C1—C2—C3	120.22 (14)	O2—C14—C5	121.13 (12)
C1—C2—H2	119.9	O2—C14—C15	119.47 (13)
C3—C2—H2	119.9	C5—C14—C15	119.40 (12)
C4—C3—C2	119.82 (15)	C24—C15—C16	120.76 (14)
C4—C3—H3	120.1	C24—C15—C14	118.79 (14)
C2—C3—H3	120.1	C16—C15—C14	120.31 (13)
C3—C4—C5	120.67 (14)	C15—C16—C17	122.72 (13)
C3—C4—H4	119.7	C15—C16—C21	118.30 (14)
C5—C4—H4	119.7	C17—C16—C21	118.98 (14)
C4—C5—C6	119.25 (13)	C18—C17—C16	120.26 (16)
C4—C5—C14	120.77 (12)	C18—C17—H17	119.9
C6—C5—C14	119.89 (12)	C16—C17—H17	119.9
C1—C6—C5	119.07 (14)	C17—C18—C19	119.9 (2)
C1—C6—C7	117.76 (12)	C17—C18—H18	120.1
C5—C6—C7	123.08 (12)	C19—C18—H18	120.1
O1—C7—C8	121.65 (14)	C20—C19—C18	120.89 (18)
O1—C7—C6	120.12 (13)	C20—C19—H19	119.6
C8—C7—C6	117.94 (11)	C18—C19—H19	119.6
C9—C8—C13	119.01 (14)	C19—C20—C21	120.97 (18)
C9—C8—C7	121.67 (13)	C19—C20—H20	119.5
C13—C8—C7	119.28 (13)	C21—C20—H20	119.5
C10—C9—C8	120.02 (15)	C20—C21—C16	119.00 (19)
C10—C9—H9	120.0	C20—C21—C22	122.68 (17)
C8—C9—H9	120.0	C16—C21—C22	118.31 (16)
C11—C10—C9	120.49 (16)	C23—C22—C21	121.63 (15)
C11—C10—H10	119.8	C23—C22—H22	119.2
C9—C10—H10	119.8	C21—C22—H22	119.2
C10—C11—C12	119.83 (16)	C22—C23—C24	120.17 (18)
C10—C11—H11	120.1	C22—C23—H23	119.9
C12—C11—H11	120.1	C24—C23—H23	119.9
C11—C12—C13	120.28 (16)	C15—C24—C23	120.82 (18)
C11—C12—H12	119.9	C15—C24—H24	119.6
C13—C12—H12	119.9	C23—C24—H24	119.6
			
C6—C1—C2—C3	−1.1 (2)	C6—C5—C14—O2	11.4 (2)
C1—C2—C3—C4	1.3 (3)	C4—C5—C14—C15	14.2 (2)
C2—C3—C4—C5	0.2 (2)	C6—C5—C14—C15	−169.37 (12)
C3—C4—C5—C6	−1.9 (2)	O2—C14—C15—C24	−87.50 (19)
C3—C4—C5—C14	174.57 (14)	C5—C14—C15—C24	93.24 (17)
C2—C1—C6—C5	−0.6 (2)	O2—C14—C15—C16	88.28 (18)
C2—C1—C6—C7	176.10 (14)	C5—C14—C15—C16	−90.98 (17)
C4—C5—C6—C1	2.1 (2)	C24—C15—C16—C17	−179.91 (15)
C14—C5—C6—C1	−174.38 (13)	C14—C15—C16—C17	4.4 (2)
C4—C5—C6—C7	−174.44 (13)	C24—C15—C16—C21	0.9 (2)
C14—C5—C6—C7	9.1 (2)	C14—C15—C16—C21	−174.84 (13)
C1—C6—C7—O1	64.4 (2)	C15—C16—C17—C18	−177.89 (15)
C5—C6—C7—O1	−118.96 (16)	C21—C16—C17—C18	1.3 (2)
C1—C6—C7—C8	−109.45 (15)	C16—C17—C18—C19	−1.0 (3)
C5—C6—C7—C8	67.15 (18)	C17—C18—C19—C20	0.1 (3)
O1—C7—C8—C9	−163.01 (15)	C18—C19—C20—C21	0.5 (3)
C6—C7—C8—C9	10.8 (2)	C19—C20—C21—C16	−0.1 (3)
O1—C7—C8—C13	14.4 (2)	C19—C20—C21—C22	178.40 (18)
C6—C7—C8—C13	−171.76 (13)	C15—C16—C21—C20	178.49 (15)
C13—C8—C9—C10	0.3 (2)	C17—C16—C21—C20	−0.8 (2)
C7—C8—C9—C10	177.73 (14)	C15—C16—C21—C22	−0.1 (2)
C8—C9—C10—C11	0.2 (2)	C17—C16—C21—C22	−179.36 (14)
C9—C10—C11—C12	0.1 (3)	C20—C21—C22—C23	−178.77 (18)
C10—C11—C12—C13	−0.7 (3)	C16—C21—C22—C23	−0.2 (3)
C11—C12—C13—C8	1.2 (3)	C21—C22—C23—C24	−0.2 (3)
C9—C8—C13—C12	−0.9 (2)	C16—C15—C24—C23	−1.3 (3)
C7—C8—C13—C12	−178.45 (14)	C14—C15—C24—C23	174.46 (16)
C4—C5—C14—O2	−165.07 (14)	C22—C23—C24—C15	1.0 (3)

### Hydrogen-bond geometry (Å, º)

Cg1 and Cg2 are the centroids of the C1–C6 and C8–C13 rings, respectively.

**Table:**

*D*—H···*A*	*D*—H	H···*A*	*D*···*A*	*D*—H···*A*
C11—H11···*Cg*1^i^	0.93	2.71	3.618 (19)	163
C20—H20···*Cg*2^ii^	0.93	2.85	3.67 (3)	141

Symmetry codes: (i) *x*−1/2, −*y*−1/2, *z*−1/2; (ii) *x*−1/2, −*y*−1/2, *z*−3/2.
